# Receptor Binding, Functional Activity, and Cell Viability Assessment of Novel Marine-Based Hybrid Peptides from *Raja porosa*

**DOI:** 10.3390/md24050181

**Published:** 2026-05-16

**Authors:** Marta Bauer, Łukasz Szeleszczuk, Bharath Kumar Velmurugan, Shang-Lun Chiang, Anna K. Laskowska, Dariusz M. Pisklak, Edina Szűcs, Dávid Gombos, Wojciech Kamysz, Tamás Fehér, Natalia Pielaszkiewicz, Krystian Małek, Patrycja Kleczkowska

**Affiliations:** 1 Department of Analytical Chemistry, Faculty of Pharmacy, Medical University of Gdansk, 80-416 Gdansk, Poland; marta.bauer@gumed.edu.pl; 2 Chair and Department of Physical Pharmacy and Bioanalysis, Department of Physical Chemistry, Faculty of Pharmacy, Medical University of Warsaw, 02-093 Warsaw, Poland; lszeleszczuk@wum.edu.pl (Ł.S.); dpisklak@wum.edu.pl (D.M.P.); 3 Maitocon Laboratory, Tirupur 641604, India; bharathvel@gmail.com; 4 Department of Medical Laboratory Science, College of Medical Science and Technology, I-Shou University, Kaohsiung 824005, Taiwan; chimpanzee99999@gmail.com; 5 Department of Pharmaceutical Microbiology, Medical University of Warsaw, 02-106 Warsaw, Poland; anna.laskowska@wum.edu.pl; 6 Institute of Biochemistry, HUN-REN Biological Research Centre, Temesvári Krt. 62, H-6726 Szeged, Hungary; szucsedina7@gmail.com (E.S.); gombos.david@gmail.com (D.G.); feher.tamas@brc.hu (T.F.); 7 Doctoral School of Theoretical Medicine, Faculty of Medicine, University of Szeged, Dóm Tér 10, H-6720 Szeged, Hungary; 8 Department of Inorganic Chemistry, Faculty of Pharmacy, Medical University of Gdansk, 80-416 Gdansk, Poland; kamysz@gumed.edu.pl; 9 Maria Sklodowska-Curie Medical Academy in Warsaw, 03-411 Warsaw, Poland; npielaszkiewicz@gmail.com; 10 Department of Biomedical Research, National Medicines Institute, 00-725 Warsaw, Poland; krystianmalekkr@gmail.com; 11 Department of Nursing and Other Health Professions, Center of Postgraduate Medical Education, 01-826 Warsaw, Poland

**Keywords:** hybrid peptide, efficacy, cytotoxicity, marine peptides, molecular target

## Abstract

The hybrid approach remains a compelling strategy for designing molecules that combine enhanced biological activity with a favorable safety profile. Marine peptides, in particular, have attracted significant attention due to their well-documented broad spectrum of biological activities. Peptides derived from rays have been recognized for their diverse biological activities. Notably, physicochemical properties of these peptides support practical application without requiring further refinement of the mature molecule or specialized formulations. In this study, we present two new chimeric peptides, PK01# and PK02#, which incorporate an opioid pharmacophore linked to a short amino acid sequence derived from the skate *Raja porosa*. Those compounds interact with the opioidergic system, specifically targeting the mu-opioid receptor (MOR). Furthermore, the compounds were evaluated for their effects on cancer cell viability through in vitro MTT assays (as an exploratory endpoint) and for their binding compatibility with EGFR via in silico docking. Both compounds showed limited effects on cell viability in HeLa, SAS, and PANC-1 cells, while PK02# induced a minor reduction in metabolic activity in glioblastoma cells without reaching IC50 values or significant cytotoxic thresholds. Interestingly, the structures of these hybrid compounds offer valuable insights into the role of phenylalanine residues within their sequences, which appear to be critical for both biological activity and receptor interaction. Moreover, these findings may support future structural optimization of peptide hybrids focused on receptor modulation and biological profiling.

## 1. Introduction

Cancer remains one of the most frequent diseases, impacting millions of individuals globally. Although there has been great progress in its diagnosis and therapy, there is still the need for improvement, as existing medicines do not offer complete protection against the disease and have substantial adverse effects. Furthermore, resistance to drugs can develop in a variety of tumors, resulting in recurrence or relapse. Thus, despite advances, cancer treatment remains only partially successful.

One way to delay or possibly overcome this challenge is to treat cancer patients with medication combinations characterized by different molecular mechanisms. However, this might encounter other difficulties that are closely related to polypharmacotherapy. Indeed, a mixture of drugs is not without side effects, the majority of which are caused by drug–drug interactions. These can be seen at both the pharmacokinetic level (i.e., reduction or augmentation of absorption, metabolism, and CYP450 enzyme interaction, etc.) and at the pharmacodynamic level (i.e., inhibition of one drug by another) [1]. Therefore, a completely different approach should be utilized in this field, which involves the use of potentially active structures/drugs but combined into a single entity, a procedure known as molecular hybridization.

The hybrid technique is a recognized strategy to enhance the biological activity of the drug while reducing the likelihood of toxicity and drug resistance as compared to that of parent compounds. A hybrid design is particularly attractive when each parent motif contributes complementary properties, such as receptor selectivity, signaling efficacy, or cellular penetration. Importantly, in the case of cancer, several compounds of this type have been shown to be efficacious [2,3,4,5,6,7]. However, to date, no rationally designed hybrid small-molecule anticancer drugs, defined as single chemical entities integrating two pharmacophores within one molecular scaffold, have been approved by the Food and Drug Administration (FDA).

**Figure 1:**
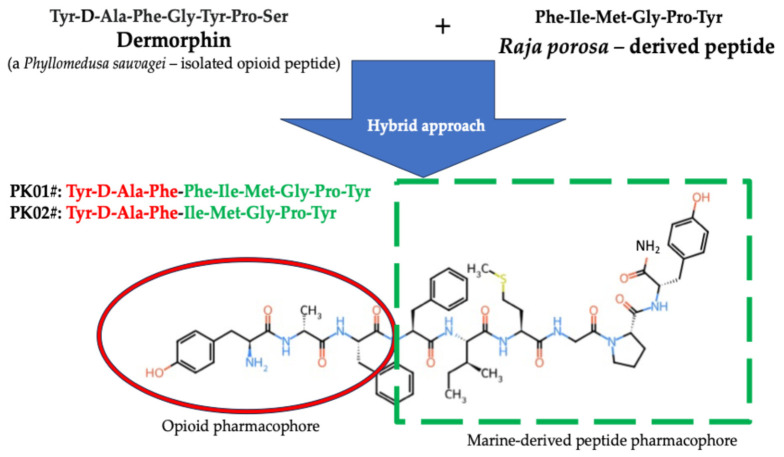
Chemical structures of the hybrid peptides PK01# and PK02#. Those hybrids were formed by linking a dermorphin fragment of a tripeptide (YdAF; Tyr-D-Ala-Phe) with a marine peptide (entire sequence of FIMGPY; Phe-Ile-Met-Gly-Pro-Tyr). The opioid pharmacophore was placed at the N-terminus to maximize affinity for the opioidergic system. In PK02#, the single Phe was removed as compared to PK01#.

Recently, marine-based peptides have attracted considerable attention due to their rich structural diversity and broad spectrum of biological activities. Compared with many terrestrial natural products, marine peptide scaffolds often display unique amino acid compositions and diverse architectures, making them attractive building blocks for medicinal optimization [8,9,10]. Nonetheless, the number of chimeric structures made of marine compounds remains quite limited. To extend the range of potentially active compounds combining two pharmacophores, one of which is a marine peptide, we present the pharmacological profile and in vitro cytotoxic activity of two new hybrid compounds, PK01# and PK02# (Figure 1). These molecules contain an N-terminal fragment of the opioid, dermorphin, and the C-terminus of a modified/unmodified peptide obtained from the skate *Raja porosa*. This combination is well justified by the fact that (i) opioid receptors, but also ligands, have been implicated in cancer-related signaling pathways [11,12,13,14]; (ii) the test sequence of the peptide from the ray-derived peptide was reported to exhibit cytotoxic activity against selected cancer cell types [15]. In addition, a receptor binding assay was performed to prove the interaction of the peptides with the opioidergic system. Also, EGFR was included as a representative cancer-related receptor for exploratory in silico analysis.

## 2. Results and Discussion

### 2.1. Binding Characteristics and Ago-/Antagonist Behavior Determination

**Figure 2:**
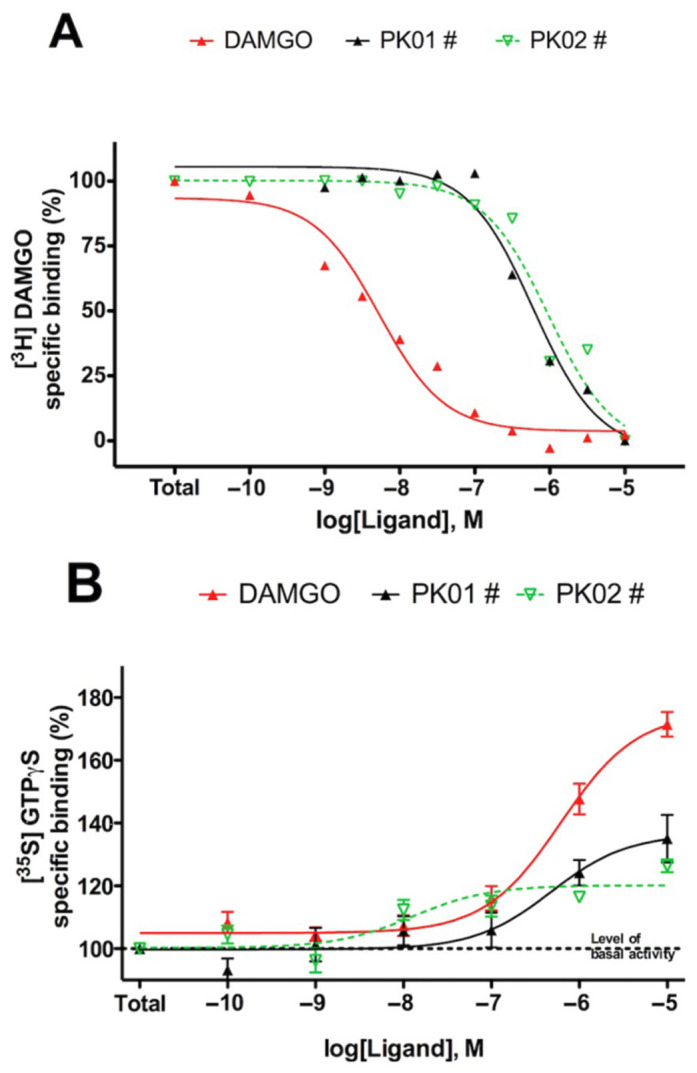
MOR binding characteristics (**A**) and G-protein activation (**B**) of PK01# and PK02#. Values represent mean values ± S.E.M. for at least three experiments performed in duplicate.

The ligands were tested for [^3^H]DAMGO homolog displacement in rat brain homogenates. Both PK01# and PK02# interacted with MOR and produced functional responses consistent with partial agonist-like behavior in the applied assay system. According to the competition binding curves (see Figure 2A), the K_i_ values for the MOR were 237.5 nM ± 15.1 nM for PK01# and 557.3 ± 128 nM for PK02#. For further investigation, the compounds were estimated for their ability to activate G-proteins using the [^35^S]GTPγS functional binding assay. PK01# and PK02# were shown to activate the G-protein with lower maximal stimulation than DAMGO (Figure 2B, Table 1), consistent with partial agonist-like activity. Emax values were 136.5 ± 5.6% for PK01#, 120.2 ± 2.3% for PK02#, and 175.2 ± 4.1% for DAMGO. Reported LogEC50 values (Table 1) were 6.3 ± 0.3 (PK01#), 7.9 ± 0.4 (PK02#), and 6.2 ± 0.1 (DAMGO). These results indicate that both hybrids preserve opioid receptor engagement, with sequence-dependent differences in affinity and signaling.

### 2.2. In Vitro Effects of PK01# and PK02# Chimeras on Cancer Cell Viability

Since opioid receptors have been shown to be expressed in various tumor cells, numerous studies suggest that they might contribute to cancer growth [16,17,18,19,20]. Moreover, other researchers have clearly demonstrated that the so-called opioid growth factor receptor (also known as zeta opioid receptor OGFr [21]), which has also been identified in several human cancer tissues, might be involved as well; its ligands were reported to inhibit cell proliferation in a variety of cancer cell lines [22,23,24,25].

As the PK01# and PK02# displayed some activity towards the opioidergic system (as presented in Figure 2), the aim of this experiment was to assess whether the receptor-active hybrid exhibits any measurable cytotoxic effects on human cervical carcinoma cells (HeLa), human glioblastoma (A-172), pancreatic ductal adenocarcinoma (PANC-1), and the cellosaurus cell line SAS. The effects of those opioid-based peptides might be mediated through opioid receptor activity. Obviously, alternative mechanisms cannot be excluded, such as interactions with non-opioid receptors such as Toll-like receptor 4, which is abundantly expressed on immune cells and certain cancer cells [26]. In contrast, FIMGPY, a hybrid pharmacophore derived from cartilage protein hydrolysates of the skate *Raja porosa* [27], has been reported to produce dose-dependent cytotoxicity in HeLa cells. Importantly, this compound was shown to be selective, targeting tumor cells while sparing normal ones [15]. Hence, evaluation of the anticancer activity of opioid/marine-derived peptide combinations appears to be well justified.

**Figure 3:**
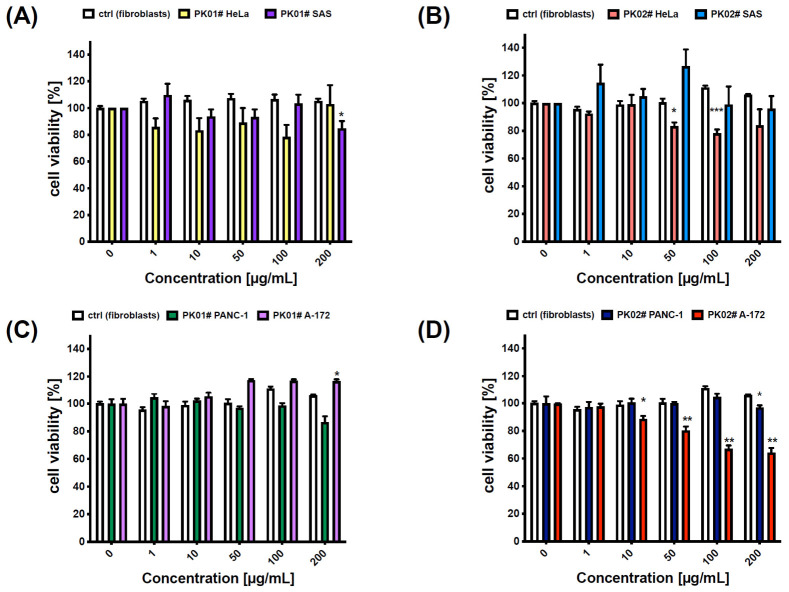
In vitro anticancer effect of PK01# (**A**,**C**) and PK02# (**B**,**D**) on HeLa, SAS, PANC-1, and A-172 cancer cell lines after a 24 h incubation. Cell viability was evaluated by MTT assay. Statistical analysis was performed with GraphPad Prism 9.0. All data are presented as mean ± S.E.M. Two-way ANOVA followed by Dunnett’s post hoc test revealed significant changes in the SAS, HeLa, PANC-1, and A-172 cells (* *p* < 0.05, ** *p* < 0.01, *** *p* < 0.001), as compared to those of control fibroblasts. IC_50_ was not determined (maximum inhibition < 50% in MTT assay).

In our study, both compounds exhibited limited effects on the tested cancer cell lines (Figure 3) across the 1–200 µg/mL concentration range. For instance, in the case of the PK01# chimera, a slight response was noticed in the A-172 cancer cell line, particularly at higher concentrations (Figure 3C), whereas almost no effect was seen in the HeLa (Figure 3A) and PANC-1 (Figure 3C) cells. In contrast, PK02# elicited more pronounced responses in HeLa and A-172 cells, suggesting a measurable effect in those lines (Figure 3B,D). Notably, human glioblastoma cell lines appeared to be the most sensitive to PK02#, with cell viability reduced to 64.33 ± 3.38% at 200 µg/mL (Figure 3D).

These findings suggest that incorporation of the dermorphin fragment (YdAF) alters the biological effect of the FIMGPY sequence—which had previously been reported by Pan and colleagues [15]—reducing its impact on cellular metabolic activity as assessed by MTT. Another plausible explanation for the lack of cytotoxic effect is the high hydrophobicity of both chimeric peptides, PK01# and PK02#. At concentrations exceeding 50 µg/mL—which, depending on molecular weight, correspond to approximately 50–100 µM—hydrophobic peptides are well known to undergo self-aggregation in aqueous media [28]. Aggregation reduces the effective free monomer concentration available for receptor interaction, limits cellular uptake, and may introduce non-specific membrane effects unrelated to the intended pharmacological targets. This is a recognized challenge in the development of hydrophobic peptide-based therapeutics and underscores the need for formulation optimization or structural modifications aimed at reducing aggregation propensity in future studies. Importantly, the additional phenylalanine residue in PK01# probably enhanced receptor affinity while showing limited effect on cell viability in the MTT assay, whereas the simplified structure of PK02# imparts partial activity in glioma cells. These observations indicate that even subtle differences in peptide sequence have an influence on the direction and extent of its biological activity.

**Figure 4:**
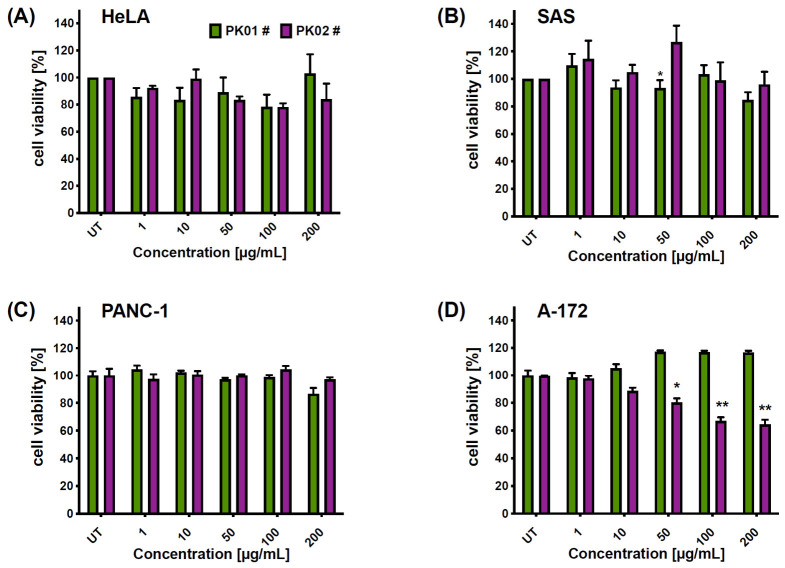
Comparison of cytotoxic effects of PK01# and PK02# after a 24 h incubation against (**A**) HeLa, (**B**) SAS, (**C**) PANC-1, and (**D**) A-172 cancer cell lines. Two-way ANOVA followed by Bonferroni’s post hoc test revealed a significant difference (* *p* < 0.05, ** *p* < 0.01) between PK01# and PK02# in SAS and A-172 cancer cells. UT denotes untreated cancer cells.

Notably, PK01# at 50 µg/mL exerted a significantly stronger activity against SAS cell lines as compared to that of PK02# (93.258% ± 5.787% vs. 126.734 ± 11.992%; Figure 4B). However, the overall compound type did not show a significant main effect on the results (F(1,14) = 1.70, *p* = 0.2134), nor was the response concentration-dependent (F(5,70) = 1.80, *p* = 0.1247). No significant differences were found in the HeLa cancer cell line, and no significant main effect of the compound occurred (F(1,6) = 0.002, *p* = 0.9585) (Figure 4A). The most pronounced reduction in cell viability was noted for the PK02# in glioblastoma (A-172), where the viability was reduced by nearly 35% at the highest concentration of the chimera (Figure 4D). Overall, cell viability effects were both compound- (F(1,4) = 153.8, *p* = 0.0002) and concentration-dependent (F(5,20) = 61.33, *p* < 0.0001). Of note, IC_50_ values could not be determined, as none of the compounds reduced cell viability by more than 50%. Also, as observed, direct comparison with classical small-molecule anticancer agents was not performed due to fundamental differences in physicochemical properties and mechanisms of action between peptide-based ligands and conventional chemotherapeutics. Peptides differ substantially from small molecules in terms of cellular uptake, stability, and intracellular distribution, which makes direct potency benchmarking between these classes methodologically limited. Therefore, the present study focuses on the intrinsic biological activity of the designed hybrid peptides rather than on comparison with standard anticancer drugs.

### 2.3. Peptide-Induced Hemolytic Effect

**Figure 5:**
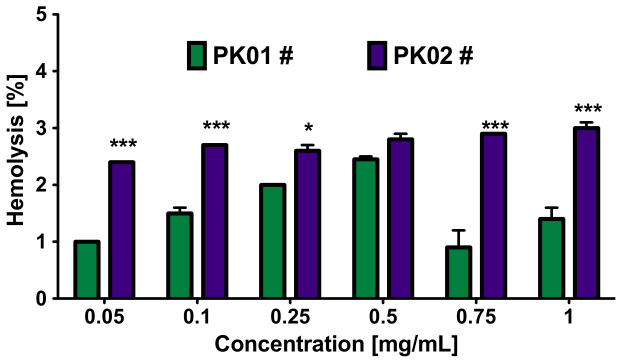
Concentration-dependent hemolytic activity of the tested hybrid compounds. Two-way ANOVA with Bonferroni’s post hoc test revealed significant differences between each concentration (* *p* < 0.05 and *** *p* < 0.005).

Both compounds showed largely neutral activity at the tested concentrations, with the exception of PK02#, which exhibited a modest effect in A-172 cells (Figure 4). This suggests that further studies on other cancer types might be desirable. At the same time, it is well known that several types of anticancer drugs have been reported to induce either immune- or non-immune-mediated acute hemolytic anemia [29,30,31,32]. Given the American Society for Clinical Pathology recommendation when the hemolysis rate is 2% or lower in laboratory blood samples [33], it is essential to identify an effective drug with a favorable safety profile, including a minimum erythrocyte toxicity. Within this context, the chimera PK01# proved to be more suitable than its analog PK02#. However, at a concentration of 0.5 mg/mL, there was no significant difference in erythrocyte damage between the two compounds (*p* > 0.05). In particular, a one-hour treatment with PK01# resulted in an acceptable hemolysis level (<2%) at all tested concentrations except for 0.5 mg/mL (Figure 5). These findings highlight the favorable erythrocyte safety profile of both chimeras, particularly PK01#. Building on this, assessment of proteolytic stability under physiological conditions represents a natural next step in their preclinical characterization, as serum protease activity during in vitro incubation may influence peptide integrity and thereby affect the interpretation of biological activity.

### 2.4. Interaction with the Opioidergic System and EGFR, as Revealed by in Silico Studies

The oral squamous cell carcinoma cell lines SAS, A-172, PANC-1, and HeLa cervical cancer cells express the epidermal growth factor receptor (EGFR) [34,35,36,37,38,39,40]. Dysregulation of EGFR is frequently observed in cancer biology, as mutation or overexpression of this protein plays a crucial role in tumor cell proliferation, survival, and metastasis [41]. Hence, EGFR was included as an exploratory in silico binding target. However, these analyses were intended to explore potential binding compatibility rather than to establish EGFR as a confirmed cellular target. In addition, we confirmed the binding affinity of PK01# and PK02# to the opioidergic system in silico.

#### 2.4.1. Molecular Docking

A comparative molecular docking analysis was performed for PK01# and PK02#, targeting the ATP-binding domain of EGFR kinase (PDB: 5D41) and the orthosteric site of the μ-opioid receptor (MOR; PDB: 5C1M). Both ligands exhibited moderately favorable docking scores in each receptor, yet a detailed interaction analysis revealed important differences in their binding patterns that might emphasize their different biological activities (Table 2).

**Figure 6:**
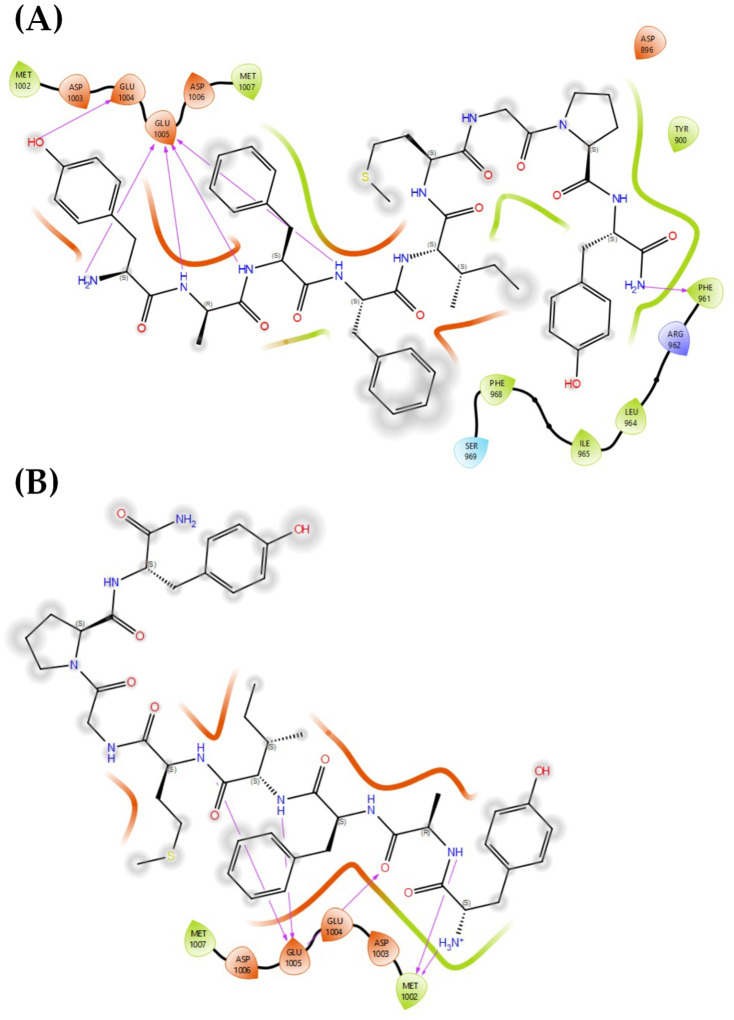
Binding site visualization of EGFR in complex with (**A**) PK01# and (**B**) PK02#.

In the case of EGFR kinase (Figure 6A), PK01# demonstrated a stronger binding affinity as suggested by its more favorable Glide score (−7.516 vs. −6.053 for PK02#). The binding mode of PK01# was stabilized by multiple polar and charged interactions, especially with residues GLU1004, GLU1005, and ASP1003, which formed well-aligned hydrogen bonds and electrostatic contacts with the amide and guanidinium groups of the ligand. In addition, MET1002 contributed to hydrophobic stabilization close to the ligand’s backbone, while π–π stacking with the central aromatic ring ultimately reinforced the interaction. In contrast, PK02# (Figure 6B), while engaging some of identical acidic residues GLU1005 and ASP1003, displayed a shifted binding pose, reducing the number of direct H-bonds and weakening the packing against MET1002. Obviously, PK02# was devoid of the salt-bridge-like interaction between its terminal amine and acidic side chains of EGFR, in contrast to PK01#.

**Figure 7:**
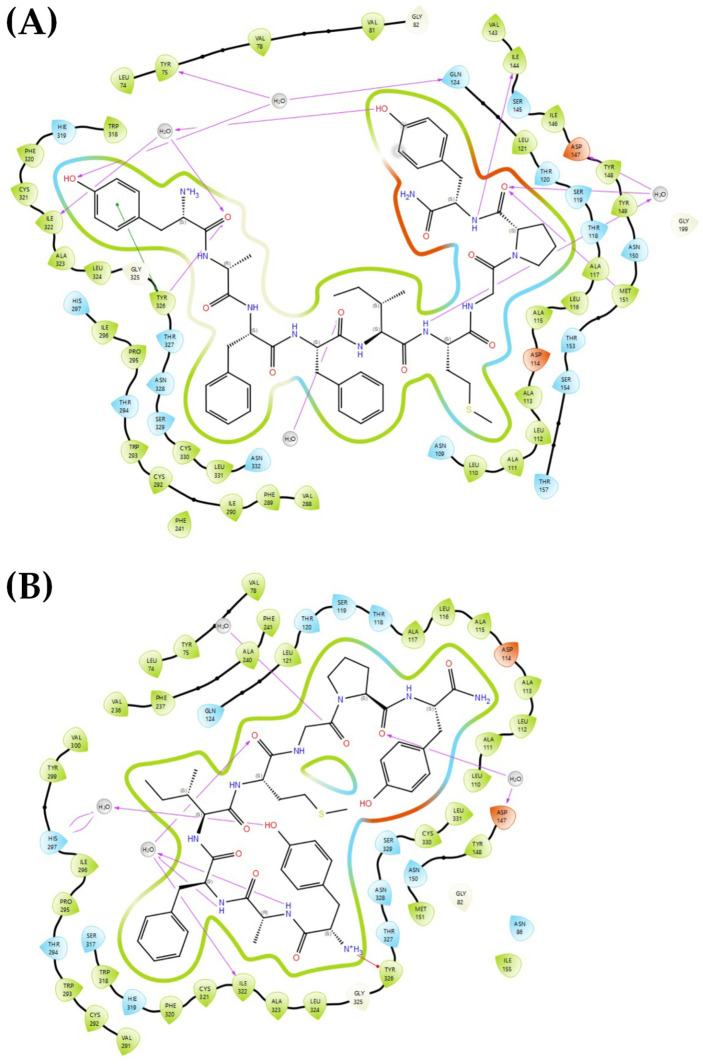
Binding site visualization of MOR in complex with (**A**) PK01# and (**B**) PK02#.

In the complex with the MOR opioid receptor (Figure 7), both PK01# and PK02# showed almost identical docking scores (~−5.6), but experimental data revealed a considerably higher affinity (Ki = 237 nM) and a stronger agonist response (E_max_ = 136.5%) of PK01# than of PK02 (Ki = 557 nM, E_max_ = 120.2%) (Figure 2, Table 1). These differences correlate well with their interaction profiles. In fact, PK01# formed a dense hydrogen bonding network with several key residues in the MOR binding pocket, including TYR75, ASP147, ILE322, and TYR326, consistent with the interaction profile summarized in Table 2. In addition, water-mediated interactions were seen with conserved residues such as TYR75 and GLN124, contributing to stabilization of its terminal phenolic group (Figure 7A). The positioning of the ligand facilitated a deeper penetration into the pocket, enhancing hydrophobic contacts with LEU1001 and MET1002.

In comparison, PK02# shared several of those polar contacts, including H-bonds with GLU1004 and GLU1005 and a water bridge to TYR75. However, it failed to reproduce the entire set of interactions characteristic of PK01#; in particular, the lack of a consistent salt bridge or water-mediated anchoring of its C-terminal group likely contributed to its lower affinity and slightly reduced efficacy. Furthermore, the positioning of PK02# appeared shallower in the pocket, thus restricting contact with deeper hydrophobic residues (Figure 7B). Interestingly, both compounds showed common interaction hotspots within each target: in EGFR, shared contacts involved ASP1003 and GLU1005, while in MOR, both ligands engaged ASP147, ILE322, and TYR326, suggesting that these residues play a central role in anchoring the ligands within their respective binding pockets. Also, both ligands used aromatic side chains to engage in π–π stacking or π–cation interactions with surrounding hydrophobic residues. However, only PK01# consistently formed extensive networks of water-mediated bridges, particularly with MOR, which likely accounts for its superior pharmacodynamic performance.

In summary, PK01# demonstrates a more robust and extensive interaction profile than PK02# does in both EGFR and MOR, with conserved polar interactions, stronger hydrogen bonding, and deeper binding site penetration. The overlap in key interactions across both targets suggests potential dual-target activity, while the discrepancies, particularly in hydration and terminal stabilization, help to explain the differences in affinity and efficacy.

#### 2.4.2. Molecular Dynamics Simulations

To verify the stability of the docking-derived complexes, molecular dynamics (MD) simulations were performed for both PK01# and PK02# in complex with two therapeutically relevant targets: the ATP-binding domain of the epidermal growth factor receptor (EGFR, PDB ID: 5D41) and the μ-opioid receptor (MOR, PDB ID: 5C1M). Each system was simulated for 100 ns under explicit solvent and physiological ionic conditions. The starting structures were the top-scoring docking poses, and the aim was to assess the conformational stability of the complexes and the persistence of key protein–ligand interactions over time.

##### Molecular Dynamics Simulations of Complexes with EGFR and MOR

**Figure 8:**
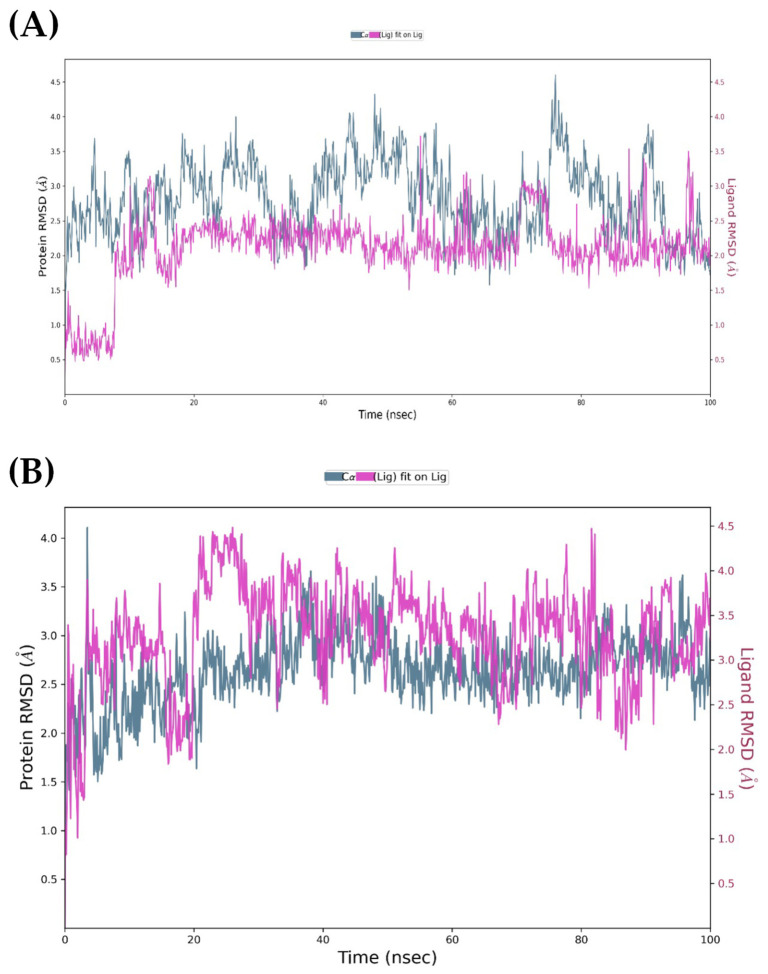
EGFR-ligand RMSD plots during 100 ns molecular dynamics simulation. (**A**): EGFR/PK01 complex; (**B**): EGFR/PK02 complex.

The backbone RMSD profiles for both EGFR runs reach a stable plateau within the first 20 ns, with fluctuations remaining in the canonical 1–3 Å window that indicates structural equilibration of a small globular protein. Visual inspection of the curves shows no monotonic drift, implying that no peptide triggers large-scale conformational change in the kinase core. Both peptides exhibited stable conformations in the binding pocket. Ligand RMSD values (Figure 8) fluctuated between 2.0 and 2.5 Å for PK01# and up to 3.0 Å for PK02# before stabilization, suggesting that neither underwent significant displacement nor unbinding.

**Figure 9:**
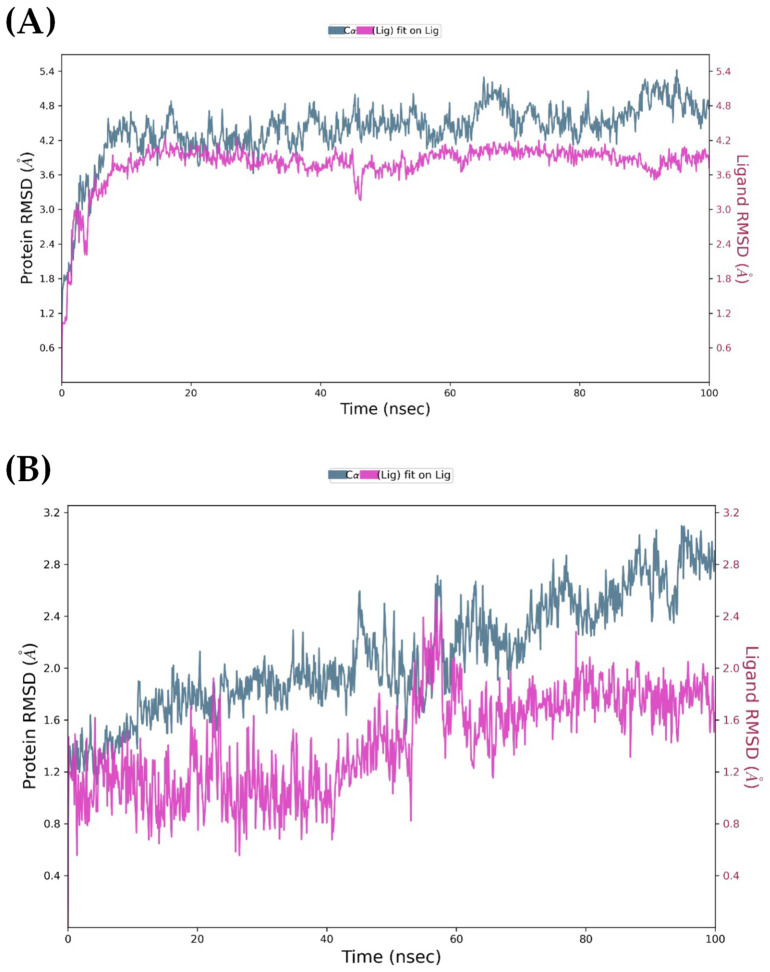
MOR-ligand RMSD plots during 100 ns molecular dynamics simulation. (**A**): MOR/PK01# complex; (**B**): MOR/PK02# complex.

The simulations of the MOR-ligand complexes (Figure 9) revealed similar trends in overall stability despite the receptor’s greater complexity and its membrane-associated nature. The protein RMSD values converged below 5 Å in both systems. The ligands remained stably embedded in the orthosteric pocket, with RMSD values around 3.5 Å for PK01# and close to 2.0 Å for PK02#, indicating robust binding throughout the 100 ns trajectories.

In conclusion, the MD simulations validated the docking results and demonstrated that PK01# and PK02# formed stable, well-defined complexes with both EGFR and MOR. The ligands remained embedded in their binding sites without significant conformational drift, preserved key interaction motifs throughout the simulations, and maintained the structural integrity of the target proteins.

Nevertheless, our results combining in silico and in vitro data suggest that although PK01# and PK02# are capable of forming stable complexes with the ATP-binding site of EGFR (favorable Glide scores, negative ΔG from MM-GBSA, and stable RMSD over 100 ns MD), translating this interaction into a broad cytotoxic effect in cells depends on additional biological conditions. The structural models adopted in this study simplify the actual context, as they do not account for receptor dimerization, phosphorylation status, the lipid environment of the membrane, receptor localization in membrane microdomains, or the presence of accessory proteins that modulate accessibility and conformation of the ATP pocket. Furthermore, the pharmacokinetic characteristics of the peptides (high hydrophobicity, the tendency to aggregate) might restrict their intracellular availability in HeLa, SAS, and PANC-1 cell lines, whereas A-172 might present favorable conditions for penetration or be more dependent on the EGFR pathway, explaining the enhanced sensitivity of this line. Alternatively, the actual effect in A-172 might be in part mediated by a different pathway or receptor (e.g., OGFr, VEGFR, TLR), and the in silico identification of interactions with EGFR reflects binding potential rather than direct evidence of a mechanism of action in cellulo. Hence, these results indicate the potential of PK hybrids to act either as receptor modulators with limited direct cytotoxicity or highlight the necessity of pharmacophore optimization to improve bioavailability and selectivity.

## 3. Materials and Methods

### 3.1. Chemicals and Reagents

Sigma-Aldrich (Budapest, Hungary) provided the following chemicals: Tris-HCl, EGTA, CaCl_2_, NaCl, MgCl_2_ × 6H_2_O, GDP, and GTP analogue GTPγS. Tyr-D-Ala-Gly-(NMe)Phe-Gly-ol (DAMGO), a highly selective MOR agonist enkephalin analogue, was obtained from Bachem Holding AG (Bubendorf, Switzerland). The non-selective opioid receptor antagonist naloxone was kindly provided by Endo Laboratories DuPont de Nemours (Wilmington, DE, USA). The ligands were dissolved in water and stored in a 1 mM stock solution at −20 °C. [^3^H]DAMGO [42] (specific activity: 36 Ci/mmol) was acquired from NOVANDI Chemistry AB (Södertälje, Sweden). The radiolabeled GTP analogue, [^35^S]GTPγS (specific activity: 1000 Ci/mmol), and the UltimaGold™ MV aqueous scintillation cocktail were obtained from PerkinElmer (Boston, MA, USA).

### 3.2. Peptide Synthesis

The PK01# and PK02# hybrid peptides were synthesized using solid-phase peptide synthesis, purified via HPLC, and confirmed by mass spectrometry.

### 3.3. Animals

Male and female Wistar rats were utilized to prepare membranes. All the animals were maintained in a temperature-controlled room (21–24 °C) with a 12:12 light–dark cycle and free access to water and food. All housing and experiments were conducted in accordance with the European Communities Council Directives (2010/63/EU) and the Hungarian Act for the Protection of Animals in Research (XXVIII.tv. 32.§). All efforts were made to minimize the number of animals used and their suffering.

### 3.4. Ex Vivo Biological Experiments

#### 3.4.1. Preparation of Brain Samples for Binding Assays

Rats were euthanized by decapitation, and their brains were rapidly extracted. Brain tissues were processed for membrane preparation following the method of Benyhe [43]. The brains were homogenized in 30 volumes (*v*/*w*) of ice-cold 50 mM Tris-HCl buffer (pH 7.4) using a Teflon-glass Braun homogenizer at 1500 rpm. The homogenate was centrifuged at 18,000 rpm for 20 min at 4 °C, the supernatant was discarded, and the pellet was taken up in the initial volume of Tris-HCl buffer. The suspension was then incubated at 37 °C for 30 min in a shaking water bath. Centrifugation was carried out again under the same conditions, and the resulting pellet was resuspended in 5 volumes of 50 mM Tris-HCl buffer (pH 7.4) and stored at −80 °C.

For [^35^S]GTPγS binding experiments, the brains were homogenized with a Dounce homogenizer in 5 volumes (*v*/*w*) of ice-cold TEM (Tris-HCl, EGTA, MgCl_2_) and stored at −80 °C. Protein concentration in the membrane fractions was determined using the Bradford assay with BSA as the reference standard.

#### 3.4.2. Competition Binding Experiments

Similar to enzymatic activity, receptor binding can be assessed through their specific ligands, allowing determination of maximal binding capacity. In competitive binding experiments, radiolabeled ligands at increasing concentrations were applied, and specific binding was quantified relative to the radioligand concentration.

In the MOR displacement assay, aliquots of the frozen rat brain membrane homogenates were thawed and resuspended in 50 mM Tris-HCl buffer (pH 7.4). The membranes were incubated with increasing concentrations (10^−10^–10^−5^ M) of unlabeled ligands at 35 °C for 45 min in the presence of [^3^H]DAMGO. The total and nonspecific bindings were determined in the absence or presence of the 10 μM unlabeled naloxone, respectively. Binding reactions were terminated by rapid vacuum filtration (Brandel M24R Cell Harvester) through glass fiber filters (Whatman GF/B for D2R and Whatman GF/C for MOR) followed by three washes with 5 mL of ice-cold 50 mM Tris-HCl (pH 7.4). The radioactivity retained on the dried filters was detected using an UltimaGold™ MV aqueous scintillation cocktail and a Packard Tricarb 2300TR liquid scintillation counter. All competitive binding experiments were performed in duplicate and independently repeated at least three times.

#### 3.4.3. Functional GTPγS Binding Stimulation Assay

Functional [^35^S]GTPγS binding assays were conducted as described previously with minor adjustments. Briefly, membrane homogenates were incubated at 30 °C for 60 min in Tris-EGTA buffer (50 mM Tris-HCl, 1 mM EGTA, 3 mM MgCl_2_, and 100 mM NaCl, pH 7.4) containing 20 MBq/0.05 cm^3^ [^35^S]GTPγS (0.05 nM) and increasing concentrations of the test ligands (10^−10^–10^−5^ M). Experiments were performed in the excess GDP (30 µM) in a final volume of 1 mL. The total binding was measured in the absence of test compounds; nonspecific binding was determined in the 10 µM unlabeled GTPγS and subtracted from total values to yield basal activity. The incubation was terminated by rapid vacuum filtration (Brandel M24R Cell Harvester), and filters (Whatman GF/B) were washed three times with 5 mL of ice-cold 50 mM Tris-HCl (pH 7.4). The radioactivity was quantified using UltimaGold™ MV scintillation cocktail and a Packard Tricarb 2300TR liquid scintillation counter. Each [^35^S]GTPγS binding experiment was performed in triplicate and repeated at least three times.

### 3.5. In Vitro Anticancer Activity Determination

#### 3.5.1. Cell Lines

The cellosaurus cell line SAS, human glioblastoma (A-172), pancreatic ductal adenocarcinoma (PANC-1), and HeLa human cervix adenocarcinoma cells were purchased from ATCC (Manassas, VA, USA). Fibroblasts were used as a control. The cells were cultured in the Minimum Essential Medium (MEM; Life Technologies, Grand Island, NY, USA) with 10% FBS (Thermo Fisher Scientific, Waltham, MA, USA) and 1% penicillin/streptomycin (Hyclone) in a humidified atmosphere at 37 °C with 5% CO_2_.

#### 3.5.2. Cytotoxicity Assay—MTT Assay

The MTT assay is a colorimetric procedure for assessing cell viability. Briefly, the cells were seeded at 5 × 10^3^ per well in 96-well plates in MEM with fetal bovine serum and incubated overnight before treatments. They were subsequently treated with a serial concentration of PK01# and PK02# (0, 1, 10, 50, 100, and 200 µg/mL) for 24 h at 5% CO_2_ at 37 °C. Cell viability (%) was assessed using an in vitro toxicology assay kit (MTT-based, Sigma-Aldrich). Cell viability was calculated by measuring the absorbance at 570 nm using a microplate reader (BioTek; Winooski, VT, USA) and comparing them with cells used as a control.

### 3.6. Hemolysis Assay

Defibrinated sheep blood was obtained from BioMaxima, (Lublin, Poland). In total, 10 mL of blood was transferred to a centrifuge tube (15 mL) and centrifuged at 2000 rpm for 10 min at room temperature (RT). The supernatant was carefully aspirated without disturbing the red blood cell (RBC) pellet. The cells were resuspended in PBS (pH 7.4, RT) and centrifuged again. The washing step was repeated four times until the supernatant appeared clear. Following the final wash, the RBC pellet was diluted 1:50 in PBS to prepare a 2% RBC suspension. Distilled water was used as a positive control (100% hemolysis), while PBS served as the negative control (0% hemolysis).

The 2% RBC suspension was then mixed with serial dilutions of the tested compounds (0.05–1 mg/mL) in a 1:1 ratio and incubated for 1 h at 37 °C. After incubation, the samples were centrifuged at 4000 rpm for 5 min at RT, and 100 µL of the supernatant from each tube was transferred into a 96-well plate. Optical density (OD) was recorded at 540 nm using a microplate reader. The percentage of compound-induced hemolysis was calculated from to the formula: Hemolysis [%] = (Ab − Ab0%)/(Ab100% − Ab0%) × 100%, where Ab = absorbance of the compound-treated sample, Ab100% = absorbance of the positive control, and Ab0% = absorbance of the negative control (PBS-treated sample).

### 3.7. In Silico Studies

#### 3.7.1. Molecular Modeling

Molecular docking, MM-GBSA calculations, and molecular dynamics (MD) simulations were performed using different modules of the Schrödinger Maestro 12.8. version (Schrödinger, LLC., New York, NY, USA, 2023).

##### Structures Preparation

Three-dimensional crystal structures of a human-active μ-opioid receptor (MOR), bound to the agonist BU72 at 2.07 Å resolution (PDB ID: 5C1M), and EGFR kinase in complex with a selective inhibitor at 2.31 Å resolution (PDB ID: 5D41) were retrieved from the RCBS PDB database. Those structures were processed using the Protein Preparation Wizard before docking to remove useless water molecules, metal ions, and cofactors. This procedure also simplifies multimeric complexes, creates disulfide bonds, assigns bond orders properly, adjusts ionization states, and fixes the orientation of misoriented groups. Hydrogen atoms were added to the protein structures, and standard protonation states at pH 7.0 were used. The preprocessed structures were then optimized and minimized to generate geometrically stable structures. The prepared protein structures were then used for further modeling.

##### Protein–Peptide Docking

The mass center of the co-crystallized ligand (BU72 or (2R)-2-(1-oxo-1,3-dihydro-2H-isoindol-2-yl)-2-phenyl-N-(1,3-thiazol-2-yl)acetamide) constituted the grid center in each case. A cubic search box was defined with the grid size set large enough to fully accommodate the peptide of 10 residues. Receptor grids were generated with the default parameters for the van der Waals scaling factor (1.00) and charge cutoff (0.25) employing the OPLS 2005 force field.

The two studied peptides, PK01# and PK02#, were prepared for docking using LigPrep from the Schrödinger Suite; protonation states were generated at pH 7.4 +/− 2.0 retaining the chiralities. For the geometry optimization, the OPLS 2005 forcefield was used. Other settings of LigPrep remained at default values.

Flexible protein–peptide docking was performed using the Grid-based Ligand Docking with Energetics (Glide) module and SP-peptide precision scheme. Glide has a special peptide docking mode (SP-peptide) designed to handle the much greater flexibility of peptides relative to the usual ligands. This mode has enhanced sampling and other settings, enabling it to capture a wider range of poses. The docking, the default 20 poses for the initial Glide docking stage, was retained. Glide score (GScore) was calculated as GScore = 0.065 ∗ vdW + 0.130 ∗ Coul + Lipo + Hbond + Metal + BuryP + RotB + Site, wherein vdW: van der Waals energy; Coul: Coulomb energy; Lipo: Lipophilic term; Hbond: Hydrogen-bonding; Metal: Metal-binding term; BuryP: Buried Polar groups’ penalty; RotB: Penalty for rotatable bonds that had been frozen; Site: active site polar interactions. Emodel combines GlideScore, the nonbonded interaction energy, and, for flexible docking, excessive internal energy of the generated ligand conformation.

#### 3.7.2. Binding Free Energy Calculation by MM/GBSA Rescoring

Calculation of the binding free energy (ΔG_bind_) was exploited to estimate in silico ligand binding affinities. The Molecular Mechanics/Generalized Born Surface Area (MM/GBSA) rescoring method was used to calculate binding free energies accurately. To do so, the Prime MM/GBSA module was used.

MM/GBSA rescoring was carried out for both the initial ligand-docked poses with the highest-scoring functions and snapshots from 100 ns MD simulation. Each MD simulation trajectory yielded 1000 images, from which average ΔGbind values were determined. The free energy changes during protein–ligand interactions were calculated using the OPLS 2005 force field and the VSGB solvent model.

The binding free energy values were calculated from the following equation:

MM/GBSA ΔG_bind_ = G_complex(optimized)_ − (G_protein(optimized)_ + G_peptide(optimized)_)

The free energy of each state, i.e., of the complex, protein, and peptide, was estimated by accounting for molecular mechanics energies, solvation energies, and entropic terms as follows: G = G_int_ + G_Coulomb_ +G_vdW_ + G_GB_ + G_lipo_ − TS, where G_int_, G_Coulomb_, G_vdW_ are standard MM energy terms for bond (covalent, angle, and dihedral), Coulomb (electrostatic), and van der Waals interactions; G_GB_ and G_lipo_ are polar and non-polar (lipophilic) contributions to the solvation free energies, while T is the absolute temperature and S is the entropy. Polar contribution (G_GB_) was calculated using the Generalized Born model, while non-polar contribution (G_lipo_) was estimated based on the solvent-accessible surface area (SASA).

#### 3.7.3. Molecular Dynamics (MD) Simulations

The obtained ligand–receptor complexes with the best docking scores constituted an input for MD simulations. For this purpose, the Desmond System Builder module was used. The systems were solvated with water, using the TIP3P water model with a buffer distance of 10 Å. The system was neutralized in each case by adding an appropriate number of Cl^−^ ions before performing MD simulations. The systems were subjected to the steepest descent minimization with Desmond’s default protocol.

The relaxation protocol consists of eight stages that include minimization with restraints on solute heavy atoms without any restraints; simulation with heating from 0 K to 300 K, H_2_O barrier, and gradual restraining; simulation under NPT equilibration with H_2_O barrier with heavy atoms restrained; NPT equilibration of solvent and lipids; simulation under the NPT ensemble with protein heavy atom restraints reduced from 10.0 to 2.0 kcal/mol; NPT equilibration with C_α_ atoms restrained at 2 kcal/mol; and simulation for 1.5 ns under the NPT ensemble with no restraints.

After relaxation, an unrestrained simulation run was performed over 100 ns for each system. The simulations were performed under the NPT ensemble using the Nose-Hoover thermostat to maintain a constant temperature of 300 K and the isotropic Martyna-Tobias-Klein barostat to maintain the pressure at 1 atm. The short-range Coulombic interactions were analyzed with a cut-off value of 9.0 Å using the short-range method. A time-reversible reference system propagator algorithm (RESPA) integrator was used with a time step of 2.0 fs. The trajectories were saved at 100 ps intervals for analysis. After simulations, RMSD, RMSF, and protein–ligand contacts were evaluated using a Simulation Interaction Diagram from the Schrödinger Suite. Interactions occurring within each frame of the performed simulations were encoded as interaction fingerprints (IFPs).

### 3.8. Data Analysis

Experimental data from ex vivo studies were presented as means ± S.E.M. Points were fitted with the professional curve-fitting program GraphPad Prism 5.0 (GraphPad Prism Software Inc., San Diego, CA, USA), using non-linear regression. In the competition binding assays, the ‘one-site competition’ fitting was used to establish the half-maximum inhibitory concentration (IC_50_ value). These values for the MOR according to the competition binding were converted into the equilibrium inhibitory constant (K_i_) using the Cheng–Prusoff equation. Inhibition was presented as a percent of the specific binding observed.

In the [^35^S]GTPγS binding assays, the sigmoidal dose–response fitting was used to establish the maximum stimulation or efficacy (Emax) of the receptors’ G-protein and the ligand potency (EC_50_). Stimulation was presented as a percent of the specific [^35^S]GTPγS binding recorded over basal activity, which was 100%.

Data from the in vitro cytotoxicity assay were expressed as the mean ± S.E.M. from three independent experiments, and GraphPad Prism 9.0 software was used to perform two-way ANOVA followed by Dunnett or Bonferroni post hoc tests. The significance level was set at *p* < 0.05.

## 4. Conclusions and Limitations

This study demonstrates that combining an opioid pharmacophore with a marine-derived peptide scaffold generates hybrid molecules with preserved μ-opioid receptor activity but limited effects on cell viability in cancer cell lines. Both PK01# and PK02# showed measurable receptor binding and partial agonist activity, while exhibiting weak cytotoxic effects across tested models.

Structural and in silico analyses confirmed stable interactions with MOR and EGFR, with PK01# showing a more extensive interaction network. However, in contrast to MOR, for which experimental validation was obtained, EGFR-related in silico observations remain purely computational and should be interpreted as hypothesis-generating only, without any implication of EGFR-mediated cellular activity.

Overall, the results indicate preserved receptor engagement with limited cytotoxic potency under the tested conditions, providing a structural basis for further optimization of peptide-based receptor ligands.

Several limitations of the present study should be acknowledged. First, the contribution of MOR-mediated signaling to the observed effects on cell viability was not directly tested using pharmacological inhibition (e.g., naloxone co-treatment) or genetic silencing of MOR in the cancer cell lines. Second, MOR expression levels were not quantified in the tested lines, which limits interpretation of receptor-dependent effects. Third, a positive cytotoxic control (e.g., doxorubicin or cisplatin) was not included in the MTT assay, which constitutes a methodological limitation, as it prevents formal validation of cell line sensitivity under the experimental conditions employed. Nonetheless, the statistical differences observed in A-172 and SAS cells (*p* < 0.05) suggest that the assay system was functional. These aspects constitute important directions for future mechanistic work.

## Figures and Tables

**Table 1:** Potency (LogEC50) and maximum effectivities of PK01# and PK02# hybrid peptides in stimulation of [^35^S]GTPγS binding in the rat brain membranes. Data are mean ± S.E.M. of 3 experiments, performed in triplicate. Maximum stimulation represents the percent stimulation of [^35^S]GTPyS binding over basal activity. The values were calculated from the dose–response binding curves shown in Figure 2B.

Ligand	Maximum Stimulation (Efficacy)	Potency
	E_max_ ± S.E.M. (%)	Log EC_50_ ± S.E.M.
DAMGO	175.2 ± 4.1	6.2 ± 0.1
PK01#	136.5 ± 5.6	6.3 ± 0.3
PK02#	120.2 ± 2.3	7.9 ± 0.4

**Table 2:** Docking scores, MM-GBSA ΔG of binding, and type of interactions in the complexes formed between the PK01# or PK02# and MOR (PDB: 5C1M) or EGFR (PDB: 5D41).

Protein	EGFR Kinase		MOR	
Ligand	PK01#	PK02#	PK01#	PK02#
Docking score	−7.516	−6.053	−5.601	−5.600
MM-GBSA [kcal/mol]	−57.4014	−27.6518	−202.9230	−147.7033
Interactions (H-bonds)	Glu1004, Asp1003, Glu1005	Met1002, Asp1003, Glu1005	Tyr75, Asp147, Ile322, Tyr326,	Asp147, His297, Ile322, Tyr326,

## Data Availability

The data are included in the text.
